# Mechanisms of generation and exudation of Tibetan medicine Shilajit (Zhaxun)

**DOI:** 10.1186/s13020-020-00343-9

**Published:** 2020-06-29

**Authors:** Rong Ding, Mingming Zhao, Jiuyu Fan, Xiuquan Hu, Meng Wang, Shihong Zhong, Rui Gu

**Affiliations:** 1 School of Pharmacy, Chengdu University of Traditional Chinese Medicine, Chengdu, 611137 China; 2 State Key Laboratory of Quality Research in Chinese Medicine, University of Macau Avenida da Universidade, Institute of Chinese Medicinal Science, Taipa, Macau 999078 China; 3 School of Energy, Chengdu University of Technology, Chengdu, 610059 China; 4 Institute of Geological Survey of Sichuan Provincial, Chengdu, 610081 China; 5 School of Pharmacy, Southwest Minzu University, Chengdu, 610041 China; 6 School of Ethnic Medicine, Chengdu University of Traditional Chinese Medicine, Chengdu, 611137 China

**Keywords:** Shilajit, Formation process, Source, Geological environment, Rock identification

## Abstract

**Background:**

Shilajit is a commonly used Tibetan medicine, and its water extract is mainly used for various heat-related syndrome, especially that of stomach, liver and kidney. Shilajit is found to exudate from rocks of cliff at an altitude of 2000–4000 m as a water-soluble mixture of black paste and animal feces of *Trodocterus* spp. *or Ochotona* spp. Because it is difficult to reach the exudation points so as to explain the its formation process, the source of Shilajit still remains unclear and controversial, which severely impedes its safety and efficacy in clinical application.

**Methods:**

In this work, a series of investigations as rock flakes identification, porosity determination, rock mineral analysis, scanning electron microscopy (SEM), and energy dispersive spectrometer (EDS) have been carried out to clarify the source of Shilajit, including the storage condition and exudation process of its organic matter, and to investigate the geological structure of the exudation points as well as physical and chemical characteristics of the mother rocks.

**Results:**

The Shilajit exudation points were mainly distributed on the steep cliffs, where there were cavities and sections that could not be eroded by rainwater. The fundamental structure of the exudation points was determined by the rock’s bedding planes, joints, fracture surfaces and faults, and developed into micro-topography later. The exudation points were distributed in the Triassic strata and scattered in the Early Mesozoic granitoids. The lithologic features were mainly slate, carbonaceous slate and sandy slate etc. The background rocks were characterized by intergranular pores, dissolved pore, joint and fracture development. Organic matter was widely distributed in these pores and fissures, which had condition for storage and exudation of organic matter.

**Conclusions:**

Shilajit mainly distributed on sunny steep slopes and cliffs with a slope of 60° or above at altitude of 2000–4000 m. The lithology character of the Shilajit exudation area were mainly various metamorphic rocks of sedimentary rocks that were rich in organic carbon. The organic matter in Shilajit was found to flow out naturally from rocks along pore, structural plane and even accumulate on the surface of rock as a result of storage environment change caused by rock tectonic action.

## Background

Shilajit, also named as Mumie, Zhaxun, is called  in Tibetan medicine, meaning the‘juice of rock’ or ‘the essence of the rock’ [1, 2]. The water extract of Shilajit is mainly used for heat related syndrome in Tibetan medicine [3]. It occupies an important position in Tibetan prescribed preparations with a rank of sixth in the most frequently used medicine [4]. The commonly used well-known prescriptions containing Shilajit include Jiu Wei Shilajit Pills, Twenty-Five Wei Yu Ganzi Pills, Zhituo Jiebai Pills and Eighteen Wei Hezi Diuretic Pills. Besides, Shilajit is also widely used by many other ethnic groups in China as well as other traditional medical systems all over the world, for example, Indian Ayurvedic medicine [5].

In China, Shilajit is mainly distributed in Aba Tibetan and Qiang Autonomous Prefecture, Ganzi Tibetan Autonomous Prefecture, Liangshan Yi Autonomous Prefecture in Sichuan Province, Bomi County in Tibet and Qinghai Province [6]. Shilajit is also widely distributed in other parts of the world [1, 7], such as the southern foothills of the Himalayas [8] (from southern Tibet in the east to Kashmir in the west), the Pamir Plateau, the Altai Mountains, the Ural Mountains [9], and the Hindu Kush [10]. It has been reported in Bhutan, Egypt, Mongolia, Nepal, India, Norway, Pakistan [11], Russia, Afghanistan, Australia [7], Tajikistan [12] and some Commonwealth of Independent States. The chemical composition of Shilajit from different regions are similar, mainly including organic matter, humic acid, fulvic acid, volatile and fat-soluble components such as taxol, verbenol, α-pinene, cypress Brain [6]. Shilajit mainly can be found on steep cliff at an altitude of 2000to 4000 m [13] and is usually mixed with animal fences, leading it difficult to study it’s source, which remains unclear and controversial.

The existing hypotheses about the source of Shilajit can be divided into two types: hypothesis of rock source and that of biological source. Scholars of both Tibetan Medicine [14] and Ayurvedic medicine [15] supported hypothesis of rock source and believed that Shilajit was a melt of metal elements such as gold, silver, copper, iron. Indian researchers [13] suggested that Shilajit was originated from marine invertebrates. Russian scholar Scholz-Böttcher reported that ‘Mumie’ was derived from the fossils of higher plants [16]. The hypothesis of biological source believed that Shilajit was derived from the dry fecal coagulum of *Trogoupterus xanthotis*, *Ochotana erythrotis*, and the fecal and urine conjugate of the squirrel [17, 18], as well as the secretions of the plant *Euphorbia royleana* Boiss., *Trifolium repens* L. and some bryophytes [10]. However, none of the current theories can either clarify clearly the source of Shilajit or can be accepted by the traditional Tibetan medicine practitioners.

The present study came up with a new hypothesis of organic matter source based on the previous research findings and the evolution rule of organic matter. Previous study showed rich organic humic acids in Shilajit presented an outflow-like characteristic in the exudation points [19]. Meanwhile, according to evolution rules [20], organic matter will pass through various stages from humic acid to kerogen, oil, natural gas and residual carbon under high temperature and pressure. Therefore, this study suggested that Shilajit was derived from the organic matter that was exuded from rock layers as a result of geological activity.

Nevertheless, to the best of our knowledge, traditional regular methods of pharmacognosy research have been unable to study the source of Shilajit. So, this study took advantages of geological research methods. Hence, a series of investigations including geological environment of the exudation points, physical and chemical characteristics of the mother rocks, storage condition and exudation process of organic matter were conducted in this paper to study the exact origin of Tibetan medicine Shilajit.

## Methods

### Research regions

Research regions in this paper covered Jinchuan County, Maerkang City, Rangtang County, Jiuzhaigou County, Aba County, Heishui County and Xiaojin County of Aba Tibetan and Qiang Autonomous Prefecture in Sichuan Province, Danba County, Daofu County, Dege County, Derong County and Baiyu County of Ganzi Tibetan Autonomous Prefecture in Sichuan Province.

### Geological environment survey of Shilajit exudation points

Route survey method [21] was used to investigate 68 Shilajit exudation points in Sichuan province of China. The elevation, terrain slope, aspect, geological structure of the Shilajit exudation position, inductive the geomorphological types, and geological structure characteristics were recorded.

### Background rock survey of the Shilajit exudation area

#### Identification of background rocks

The background rocks were identified and formation lithology and rock composition of the Shilajit exudation points were analyzed.

Appraisal basis were conducted in accordance with the China National Standard: igneous rock—GB/T 17412.1-1998 [22], classification and naming scheme of igneous rocks; sedimentary rock—GB/T 17412.2-1998 [23], classification and naming scheme of sedimentary rock; metamorphic rock—GB/T 17412.3-1998 [24], classification and naming scheme of metamorphic rock. The technical specifications used in this study include DZ/T 0275.1-2015, DZ/T 0275.4-2015 and DZ/T0130.9-2006 [25–27].

#### Determination of organic carbon and total organic carbon (TOC) content in background rocks

The rock mineral analysis method was used to determine the content of organic carbon in the background rocks and ordinary rocks of Shilajit [28]. The rock samples were heated in 10% hydrochloric acid to remove the carbonate, washed with water, and after removing the chloride ions, it was dried at 80 °C. Then the organic carbon was burned and was converted into carbon dioxide gas in a high-temperature oxygen stream, and was monitored by high frequency infrared carbon sulfur analyzer using HCS-140 system (Caide Instrument, Shanghai, China).

#### Research of background rock storage space

Several batches of background rock samples were selected, the columnar rock samples were drilled by a cutter, and the prepared blue epoxy resin was poured into the columnar rock samples under vacuum. The SMJ automatic grinding machine was used to grind the sheet and observed in 59XD polarizing microscope (Nikon, Japan).

### Background rock SEM and EDS

Conventional optical microscopes can only observe the microstructure and pore characteristics of minerals, when combined with SEM and energy spectrometer, preliminary analysis of rocks with different structural planes can be carried out [29]. Firstly, the distribution and morphological characteristics of minerals in background rocks were observed by SEM using a FEI Quanta FEG 250 system (FEI, America). The mineral composition was analyzed by EDS using an Oxford INCAx-max20 system (Oxford, England). Finally, the mineral characteristics of the background rocks and the content of mineral constituents were obtained.

There were 35 samples in total from Aba Tibetan and Qiang Autonomous Prefecture, including 13 batches of Mozigou, Danba County, 13 batches of Muerzong Township and 9 Longerjia Township, Maerkang City. First, a geological hammer was used to knock out a block rock sample with an area of about 5.2 cm; a fresh, flat natural fracture surface was selected as the observation surface, and the machine was observed after gold plating. From this, the microscopic characteristics of the bedrock and pore fillings were observed, the elemental contents of the mother rock and the filling were determined, and the properties of the pore filling and the relationship between the mother rock and the filler were found.

#### Determination of background rock porosity

The connected porosity of Shilajit background rock was determined by saturated kerosene method [30].

## Results

**Fig. 1:**
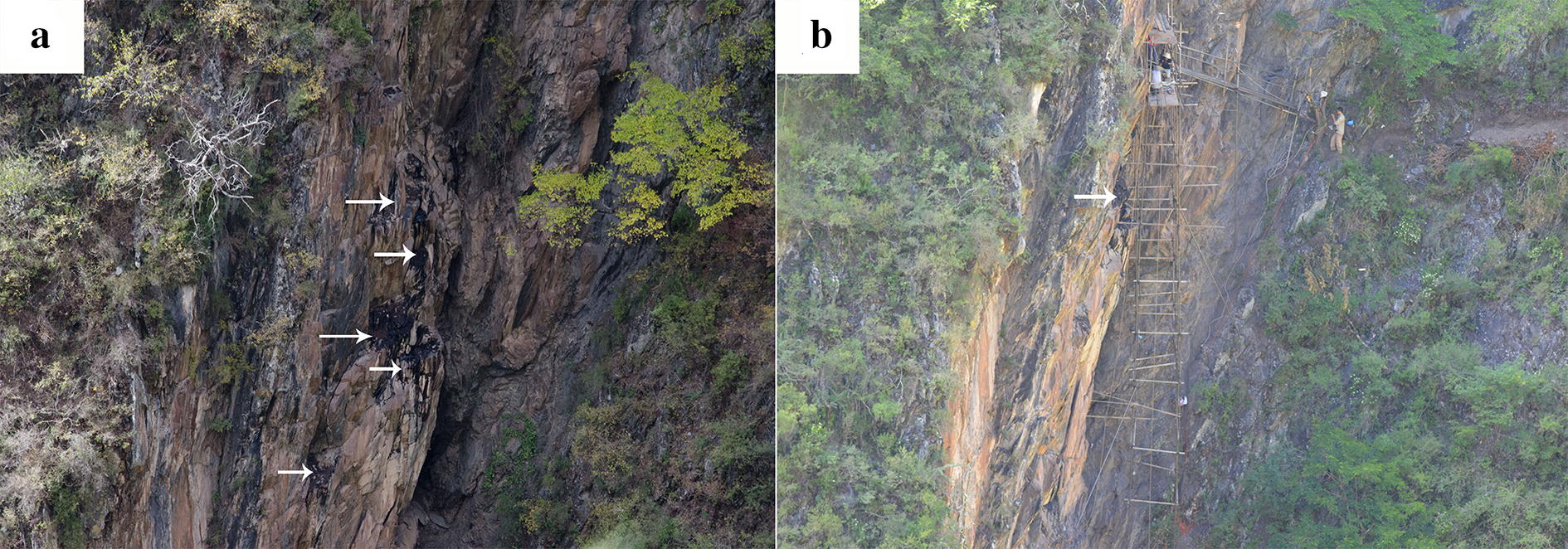
The location of the survey point and long-term observation point, white arrow pointed the point of seepage. (**a** and **b** were in the same position)

The investigation of the exudation points showed that Shilajit was mainly distributed in Duke River, Dajinchuan River, Gesheza River, Xiaojinchuan River, Dawei River, Fubian River, Jiaomuzu River, Suomo River and Heishui River in Aba Prefecture, Sichuan province, and Dingqu River and Aqu River Basin in Ganzi Prefecture. The geographical location of the Shilajit exudation points and the measurement project information of the background rocks were presented in Table 1. The location of the survey point and long-term observation point were shown in Fig. 1.

**Table 1:** Detailed information of Shilajit exudation points

No.	Autonomous Prefecture	Origin/source	Latitude	Longitude	Height	Slope/aspect	Measurement
1.	Ganzi	Donggu Township, Danba County	30.68237222	101.7433667	2569 m	S188°	Rock identification
2.	Ganzi	Donggu Township, Danba County	30.72183611	101.7448611	2567 m	E74°	Rock identification
3.	Ganzi	Mozigou, Danba County	31.06226944	101.6442028	2534 m	ES116°	Casting thin sections/Scanning electron microscopy/Energy spectrum analysis/porosity
4.	Ganzi	Mozigou, Danba County	31.05345	101.6386528	2497 m	EN51°	Casting thin sections/scanning electron microscopy/energy spectrum analysis/porosity
5.	Ganzi	Banshanmen Township, Danba County	30.99472222	102.0391667		E102°	
6.	Ganzi	Banshanmen Township, Danba County	31.00277778	102.0575		EN56°	
7.	Ganzi	Banshanmen Township, Danba County	30.98725	102.0281056		ES117°	
8.	Ganzi	Diaobao Village, Banshanmen Township, Danba County	30.99	102.0327778		EN107°	
9.	Ganzi	Waba Village, Keshenzha Township, Danba County	30.91388056	101.7671806	2108 m	E80°	
10.	Ganzi	Derong County	28.91822778	99.39025			Rock identification
11.	Ganzi	Dege County					Rock identification
12.	Aba	Shili Township, Rangtang County	31.88819722	101.1102111	2937 m	ES138°	Rock identification
13.	Aba	Shili Township, Rangtang County	31.91601389	101.0917833	3077 m	W284°	
14,	Aba	Wuyi County, Rangtang Township	32.13999722	101.00475	3150 m	WN317°	Rock identification
15.	Aba	Puxi Township, Rangtang County	31.78842222	101.2587278	2985 m	E93°	
16.	Aba	Genzha Township, Jinchuan County	31.80038889	101.9148306	2438 m	EN56°	
17.	Aba	Kalazu Township, Jinchuan County	31.640625	101.9659694	2768 m	ES137°	
18.	Aba	Kalazu Township, Jinchuan County	31.63472778	101.9666972	2697 m	E98°	
19.	Aba	Hexi Township, Jinchuan County	31.39898611	102.0405611	2138 m	WN313°	
20.	Aba	Anning Township, Jinchuan County	31.27309722	102.0410611	2081 m	N10°	
21.	Aba	Akening Township, Jinchuan County	31.989575	101.7301861	2481 m	E88°	
22.	Aba	Jimu Township, Jinchuan County	31.80039167	101.9148306	2438 m	E74°	
23.	Aba	Dusong Township, Jinchuan County	31.31391944	101.9997056	2289 m	ES144°	Rock identification
24.	Aba	Dusong Township, Jinchuan County	31.31315833	101.9993417	2211 m	EN40°	
25.	Aba	Dusong Township, Jinchuan County	31.3172	102.1554083	2145 m	N14°	
26.	Aba	Xinge Township, Xiaojin County	31.03166667	102.1677778		EN61°	
27.	Aba	Xiaojin County	31.01568889	102.3202028	2268 m	EN57°	
28.	Aba	Meiwo Township, Xiaojin County	30.922625	102.4005	2658 m	W278°	Rock identification
29.	Aba	Dawei Town, Xiaojin County	30.93343056	102.6441667		ES150°	
30.	Aba	Shuangbai Township, Xiaojin County	31.11424444	102.4336028	2488 m	W271°	
31.	Aba	Fubian Township, Xiaojin County	31.32825	102.5006139	2781 m	ES148°	Casting thin sections
32.	Aba	Meiwogou, Xiaojin County	30.93626667	102.3991806	2554 m	W269°	
33.	Aba	DabakouVillage, Xiaojin County	31.01568889	102.3202028	2268 m	EN57°	
34.	Aba	Jiaomuzu Township, Maerkang City	32.10549167	102.015075	2491 m	WN324°	
35.	Aba	Suomo Township, Maerkang City	31.87569444	102.3112472		ES128°	
36.	Aba	Longerjia Township, Maerkang City	32.17472222	101.9847222		W285°	Casting thin sections/scanning electron microscopy/energy spectrum analysis/porosity
37.	Aba	Longerjia Township, Maerkang City	32.21346389	101.9044639			Casting thin sections/scanning electron microscopy/energy spectrum analysis/porosity
38.	Aba	Muerzong Township, Maerkang City	31.84951667	101.7590278	2451 m	S181°	Casting thin sections
39.	Aba	Caodeng Township, Maerkang City	32.21424444	101.8302444			
40.	Aba	Baiwan Township, Maerkang City	31.99704167	101.830425	2393 m	EN27°	Rock identification
41.	Aba	Baiwan Township, Maerkang City	31.8415	101.7931278	2426 m	N356°	
42.	Aba	Baiwan Township, Maerkang City	31.76828611	101.9741611	2348 m	WS205°	Casting thin sections/scanning electron microscopy/energy spectrum analysis/porosity
43.	Aba	Maerkang City	31.90175278	102.2039917			Rock identification
44.	Aba	Heishui County	30.06837778	103.2245944	2229 m	W273°	Rock identification
45.	Aba	Luoduo Township, Heishui City	32.05062222	103.3426278	2798 m	N348°	
46.	Aba	Chibusu Township, Mao County	31.89866389	103.4422889	1817 m	WN336°	
47.	Tibet	Jiangda County					Rock identification

### Geological environment study of Shilajit exudation points

#### Topography and geomorphology features of the Shilajit exudation points

*Geomorphological types*-The geological survey results of 74 Shilajit exudation points indicated that they were mainly distributed on steep slopes and steep cliffs with steep terrain. The exudation points were distributed in cliff cavities and section which could not be eroded by rainwater. It was consistent with the fact that strong tectonic activity, deep valley cutting and controlled structural plane development of the Songpan-Ganzi orogenic belt were observed in Shilajit points.

*Distribution elevation*- The investigated Shilajit exudation points were mainly distributed at an elevation of 2000–4000 m. Among them, 28, 24, 13 and 9 exudation points were located at an elevation of 2000–2500, 2500–3000, 3500–4000, and 3500–4000 m respectively.

**Fig. 2:**
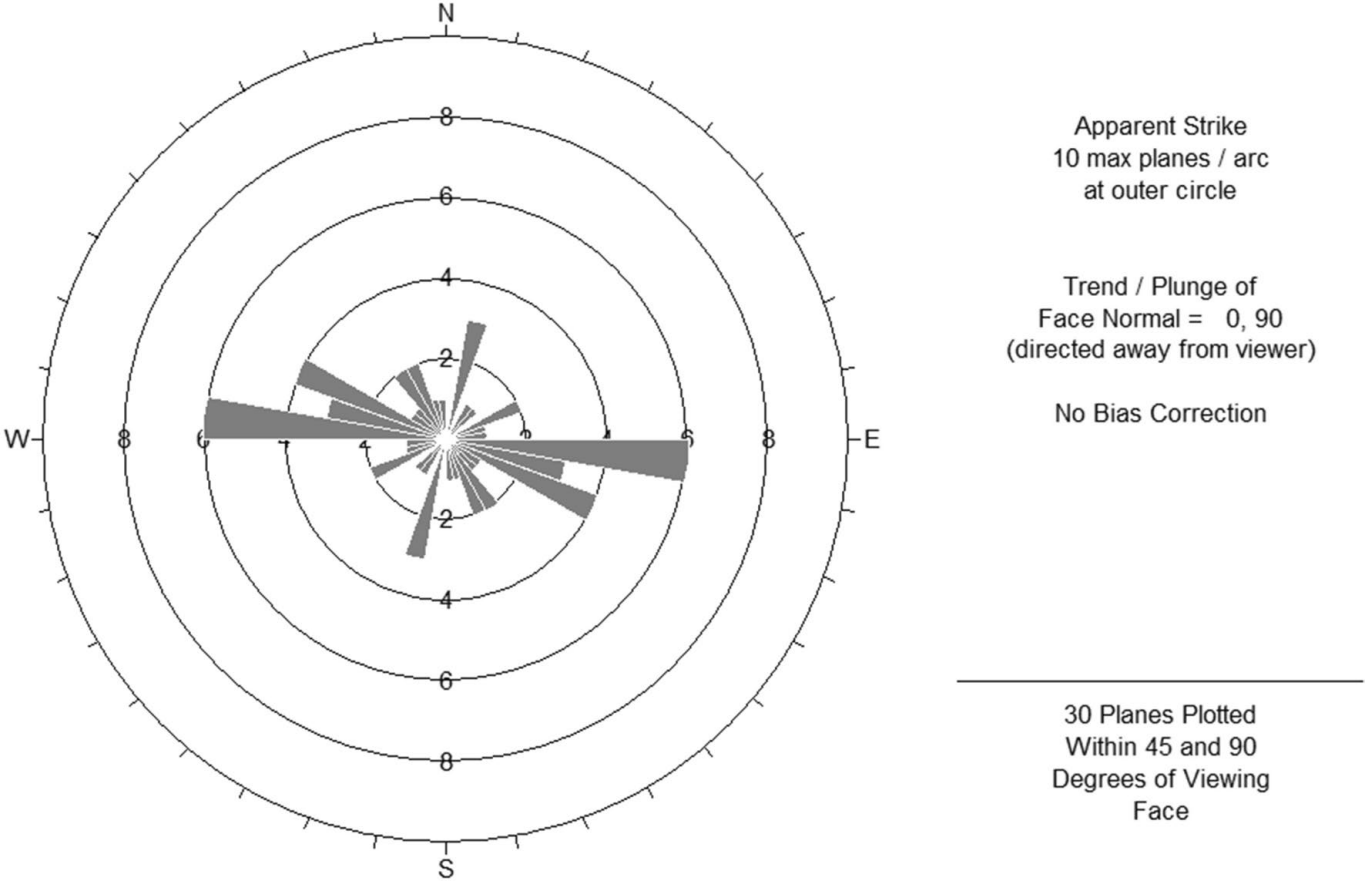
Slope feature of Shilajit exudation points

*Aspect*- Mainly four groups of dominant aspects were observed, including: 1. NW 270°–280°; 2. NW 290°–300°; 3. NE 10°–20°. As shown in Fig. 2. These slopes were all sunny slopes, indicating that Shilajit tended to be exuded from sunny slope, which was consistent with the recorded of Tibetan classic ‘Jinzhu materia medica’ [31]. This result suggested heating effects of sunlight on rocks might be responsible for the exudation of Shilajit.

#### Geological structure and control structure surface of the Shilajit exudation points

**Fig. 3:**
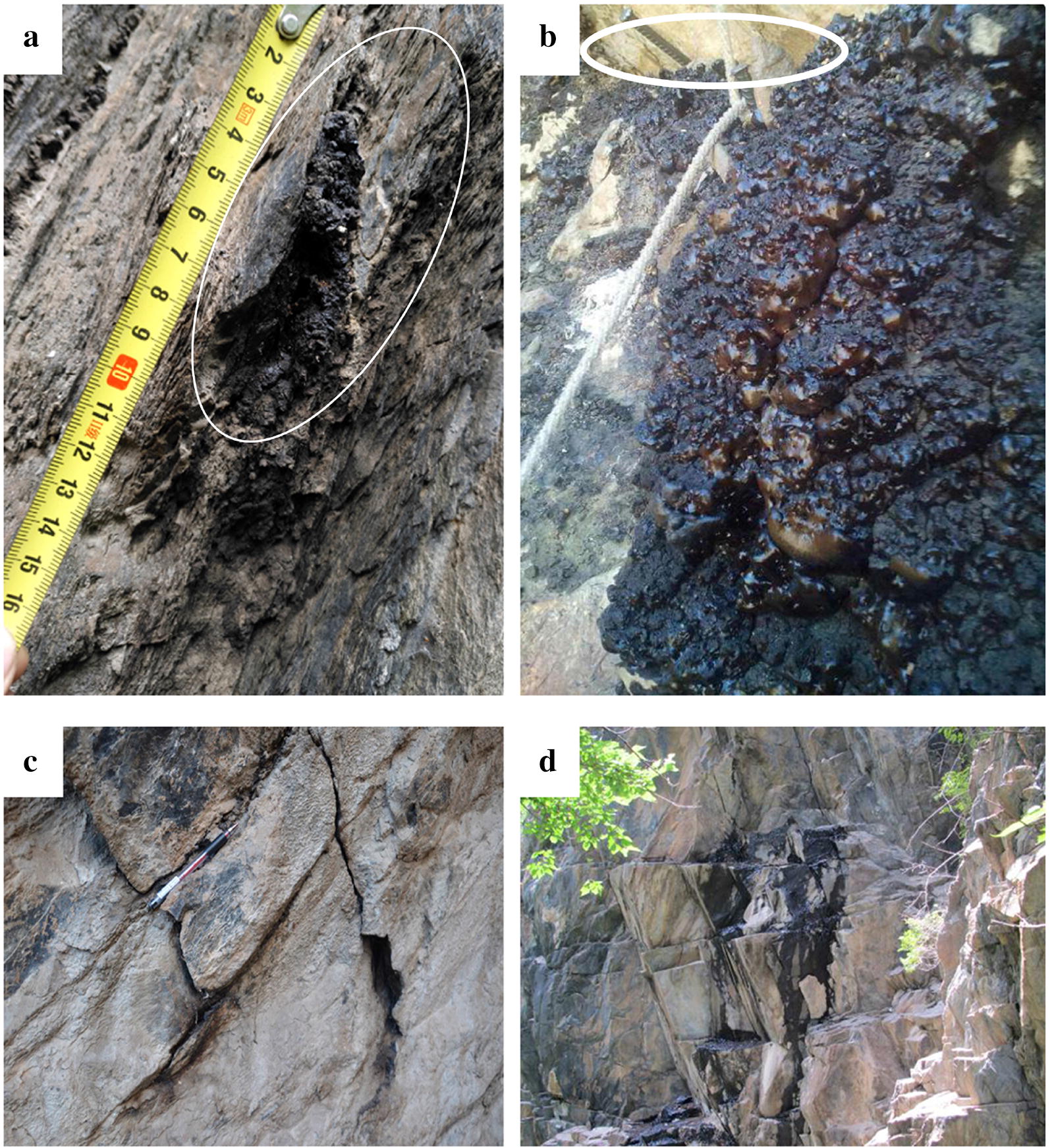
Controlled structural surface of the Shilajit exudation area (The black substance was Shilajit). **a** Fault plane. **b** Joint plane. **c** Joint plane. **d** Comprehensive structure of fault planes and joint planes
- Unloading cracks and fault control (Fig. 4), such as: the broken rocks in developed areas of folds or faults form concave cavities and holes. There were distribution points of fault planes, joint planes and the places where Shilajit could be easily seeped out.

The Shilajit exudation points were located in foreland basin of Bayan Kala-Songpan periphery in the Songpan-Ganzi orogenic belt. The foreland basin was connected to South Kunlun-Maqu-Ma-Qin belt in the north. The northeast was bounded by the Minjiang-Huya large-scale structure and the Pingwu–Qingchuan fault. South stopped at the Xianshui River fault structure and extended into Qinghai. Field geological survey results indicated that the control structural plane of Shilajit exudation location mainly included the following types:

- Rock’s own structural plane control, fault plane, joint plane, etc. (Fig. 3), consisting the basic structure of Shilajit exudation points.

**Fig. 4:**
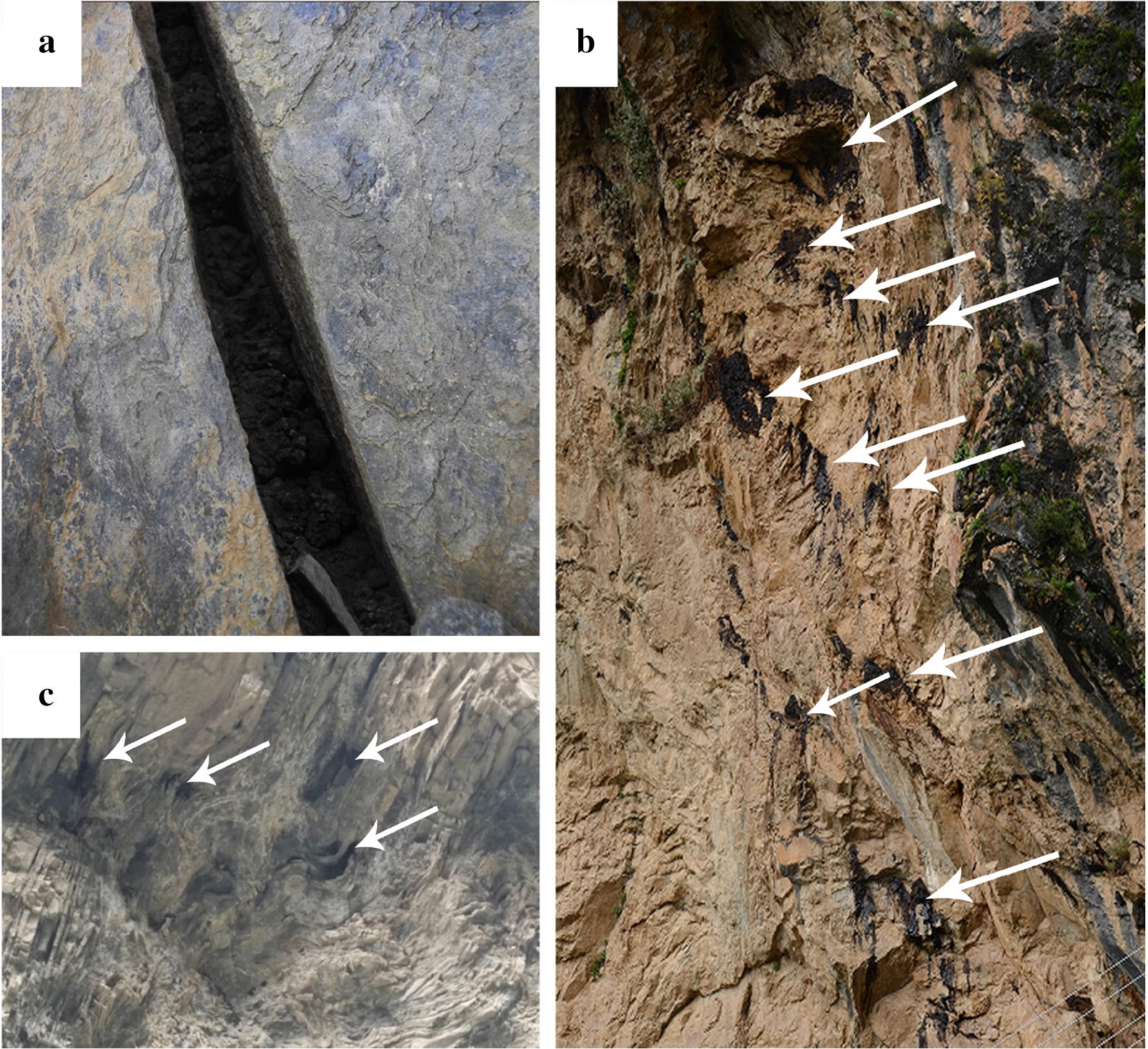
Unloading crack and fault structure of Shilajit exudation points, white arrow pointed the point of seepage. **a** Unloading crack. **b** Fault. **c** Fold
- Concave cavity, wind erosion hole, steep cliff and other microgeomorphic control structure (Fig. 5). Concave cavity was a group of fault planes or joint planes formed by the falling off of rocks due to the action of gravity. Wind erosion holes were formed by wind erosion in softer parts of the rock. These holes were often the exudation area of joints or fault planes and also the exudation points of Shilajit.

**Fig. 5:**
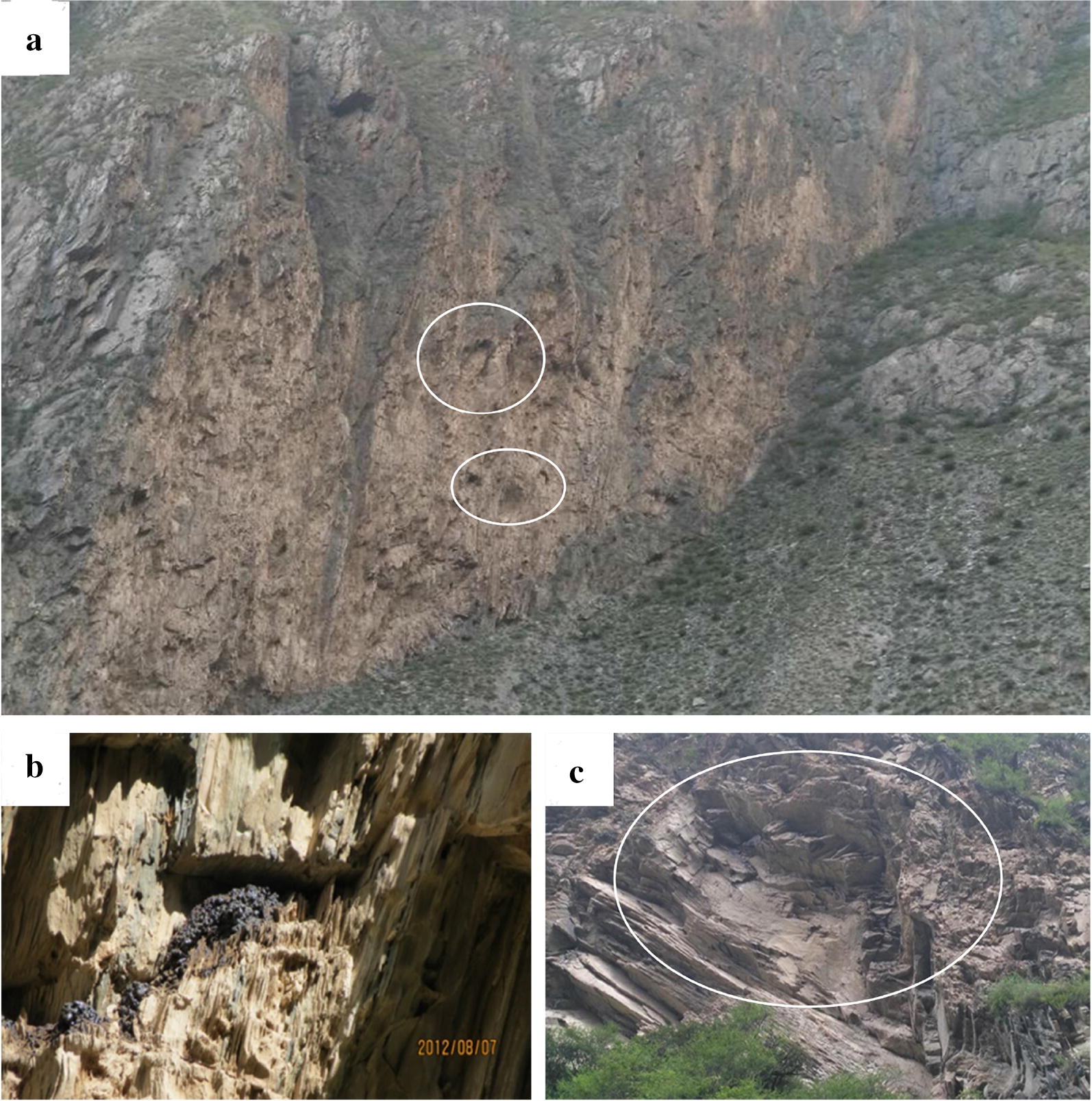
Micro-geomorphic type of Shilajit exudation points. **a** Wind erosion hole. **b** Concave cavity. **c** Concave cavity

It can be analyzed that exudation and formation of Shilajit were closely related to geological processes. Moreover, rock tectonic action led to storage environment (temperature, pressure, structural plane) changes, which in return caused the organic matter in the rock to naturally ooze along the pores, the structural surface, and even accumulate on the rock surface.

### Geological background study of Shilajit exudation area

#### Geological structure and geological history analysis of the Shilajit exudation area

**Fig. 6:**
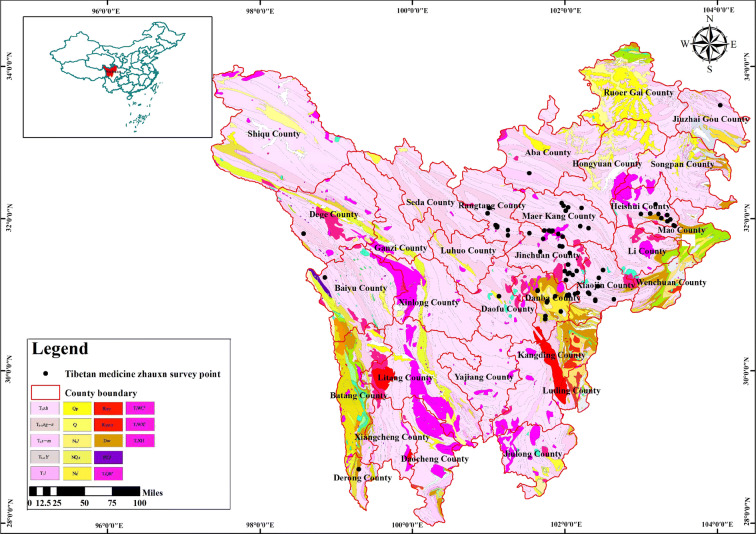
Geological sketch of the Shilajit distribution area

The strata of Shilajit exudation points were mainly distributed in Xinduqiao Formation (T_3_xd), Zhagashan + Zagunao Formation (T_2-3_zg-z), Zhuwo Formation (T_3_zh) and Yantang Formation (T_1-2_y) of the Triassic system, at the same time, there were sporadic distributions in the Early Mesozoic granites. The coordinate points and map data of Shilajit were imported into ArcGis10.5 software to generate Fig. 6, while the basic map data was provided by Institute of Geological Survey of Sichuan Provincial, China. Figure 6 showed the distribution of the Shilajit field survey points and the geological setting of the distribution area. The lithology character of T_3_xd included gray-black sericite slate, phyllite, metamorphic sandstone. The lithology character of T_3_zh included dark gray meta sandstone, sandstone and carbonaceous slate. The lithology character of T_1-2_y included dark gray bioclastic micrite.

The intrusive rocks of late Yanshanian were mainly composed of silicon, aluminum and supersaturated acid rocks. Characteristic trace elements of acid rock, such as Li, Be and Sn had higher content in these rocks, with good ore-bearing and potential mineralization prospects [32].

### Geological background study of Shilajit exudation area

#### Identification of background rocks

Geological survey results of Shilajit exudation points indicated that lithologic characteristics of exudation area were mainly slate, carbonaceous slate, sandy slate, phyllite, meta sandstone, limestone and a small amount of granite.

**Fig. 7:**
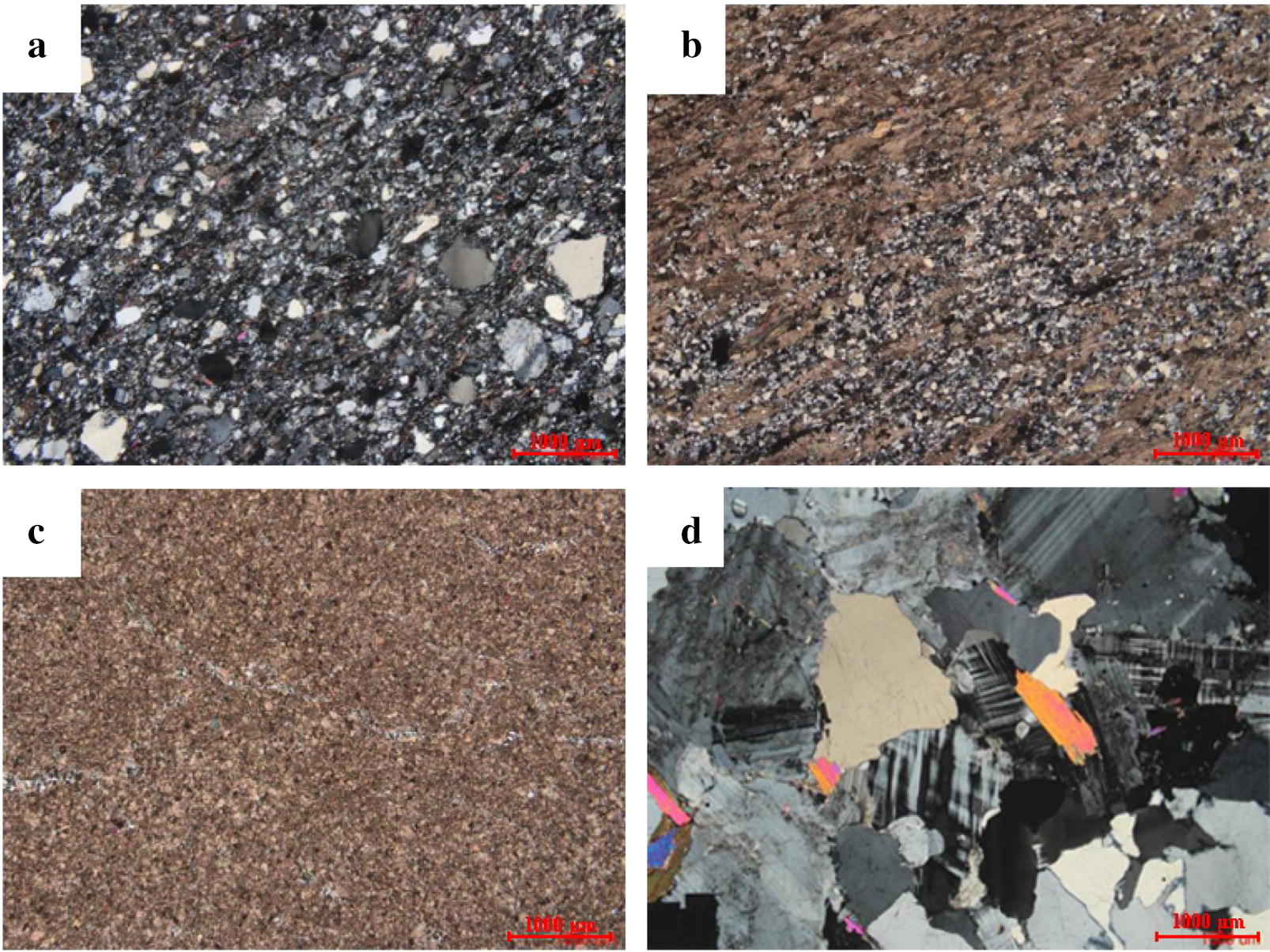
Typical results of thin section identification of Shilajit background rocks. **a** Rock samples from Longerjia Township, Maerkang City, Aba Autonomous Prefecture, Sichuan Province, anisomerous blastopsammitic texture. **b** Rock samples from Dege County, Ganzi Autonomous Prefecture, Sichuan Province, granoblastic texture. **c** Rock samples from Derong County, Ganzi Autonomous Prefecture, Sichuan Province, Aplitic texture. **d** Rock samples from Muerzong Township, Maerkang City, Aba Autonomous Prefecture, Sichuan Province, Medium-grained blastogranitic texture

The thin section identification of 17 batches of background rocks indicated that rock lithology mainly included silt-bearing fine sandstone, calcite quartz sericite phyllite, staurolite-bearing felsic sericite phyllite, metamorphic sandstone, silty metamorphic sandstone and (metamorphism) fine powder crystal dolomite. Among them, the samples lithology of Muerzong Township of Malcolm City, Mozigou of Danba County and Jiangda County of Tibet were characterized by granite (Table 2 and Fig. 7).

**Table 2:** Thin section identification of background rocks

No.	Autonomous Prefecture	Origin/source	Rock texture	Rock structure	Identification name
1.	Ganzi	Dege County	Granoblastic texture	Massive structure	Calcite quartzite
2.	Ganzi	Baisong Township, Derong County	Aplitic texture	Massive structure	(Metamorphism) Fine powder crystal dolomite
3.	Ganzi	Donggu Township, Danba County	Lepido granoblastic texture	Phyllitic structure	Calcite quartz sericite phyllite
4.	Ganzi	Danba County	Medium fine-grained blastogranitic texture	Massive structure	Metamorphic medium-fine grained two-mica adamellite
5.	Aba	Cao Deng Township, Maerkang City	Lepido granoblastic texture	Phyllitic structure	Staurolite-bearing felsic sericite phyllite
6.	Aba	Cao Deng Township, Maerkang City	Lepido granoblastic texture	Phyllitic structure	Staurolite-bearing felsic sericite phyllite
7.	Aba	Longerjia Township, Maerkang City	Anisomerous blastopsammitic texture	Oriented structure	Metamorphic sandstone
8.	Aba	Maerkang City	Aleuritic anisomerous blastopsammitic texture	Oriented structure	Silty metamorphic sandstone
9.	Aba	Baiwan Township, Maerkang City	Lepido granoblastic texture	Parallel grain structure	Sillimanite-bearing two-mica granulite
10.	Aba	Muerzong Township, Maerkang City	Medium-grained blastogranitic texture	Massive structure	Metamorphic medium-grained two-mica adamellite
11.	Aba	Maerkang City	Medium fine-grained blastopsammitic texture	Oriented structure	Metamorphic medium-fine grained lithic arkose
12.	Aba	Fubian Township, Xiaojin County	Fine-grained blastopsammitic texture	Oriented structure	Calcareous metamorphic fine sandstone
13.	Aba	Xiaojin County	Fine-grained blastopsammitic texture	Oriented structure	Calcareous metamorphic fine sandstone
14.	Aba	Dusong Township, Jinchuan County	Lepido granoblastic texture	Massive structure	Biotite granulite
15.	Aba	Shili Township, Rangtang County	Aleuritic fine-grained blastopsammitic texture	Oriented structure	Silty-bearing metamorphic fine sandstone
16.	Aba	Heishui County	Blastopsammitic texture	Platy structure	Metamorphic sandstone
17.	Tibet	Jiangda County	Fine-grained granitic texture and breccia texture	Massive structure	Brecciated tonalite

**Fig. 8:**
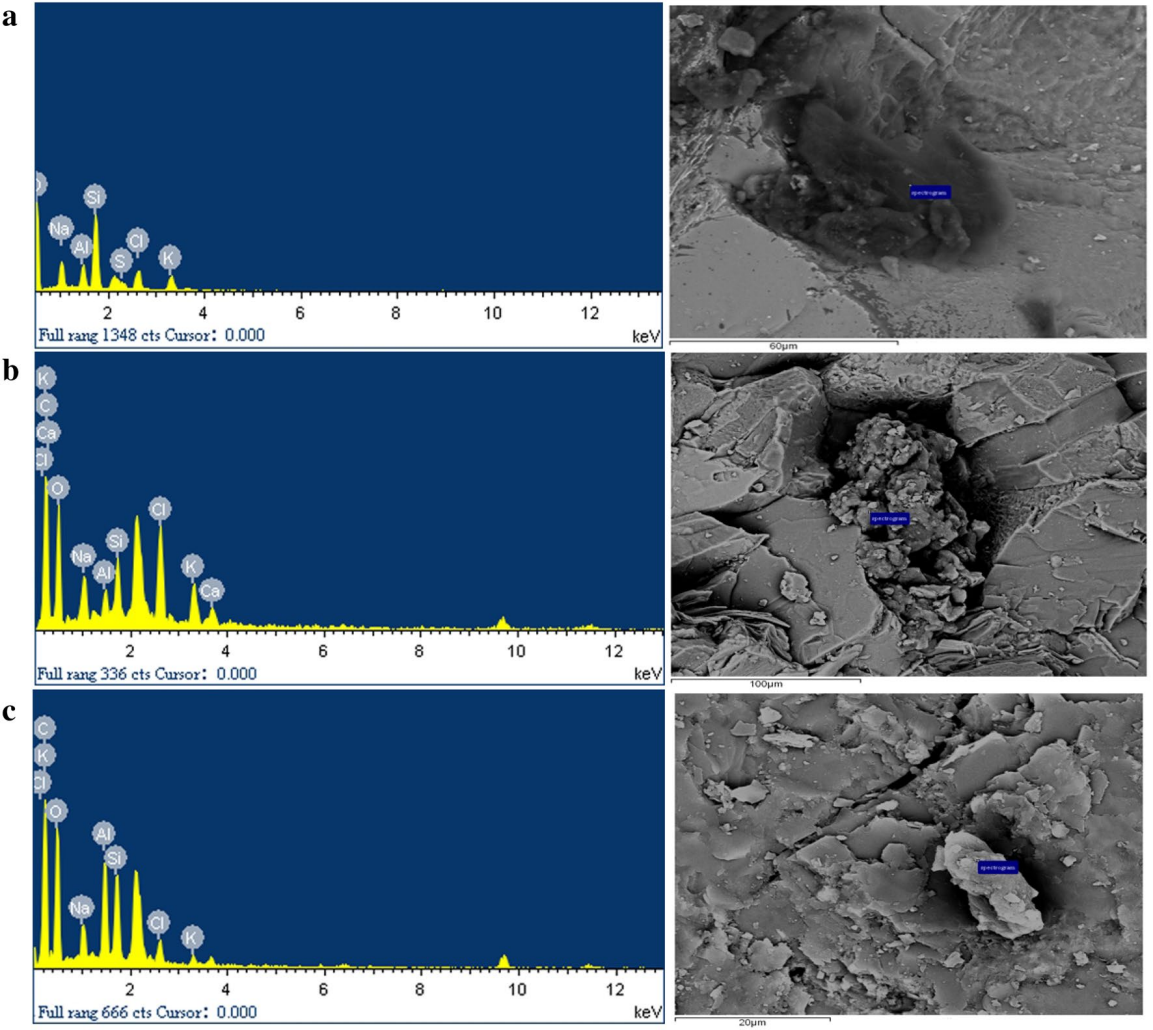
EDS results, the right picture was the analysis point

Among them, the sandstone and phyllite had high sand content. The rock may have pores, and there was possibility of storing organic matter and water. Since Shilajit can be easily dissolved in water and phyllite is a waterproof barrier, the rock showed natural condition of storing Shilajit. Because granite is an intrusive igneous rock, there is no pore development, but there may be possibility of storing organic matter in its fissures and joints.

#### Determination of organic carbon and TOC

The tests were conducted in accordance with the China National Standard DZG20.01-1991. Comparing to regular rocks, the background rocks containing Shilajit had significantly higher organic carbon content, as shown in Table 3, indicating that organic matter in Shilajit might be derived from background rocks.

**Table 3:** Organic carbon content TOC in Shilajit background rocks

Producing area	Results (%)	
	Organic carbon	TOC
Cao Deng Township, Maerkang City, Aba Prefecture B-b	0.930	1.120
Cao Deng Township, Maerkang City, Aba Prefecture E-b	0.905	1.120
Longerjia Township, Maerkang City, Aba Prefecture	1.000	1.110
Maerkang City, Aba Prefecture	0.265	0.345

### Background rock SEM and EDS

EDS of 35 samples showed that content of organic matter in pores and cracks of the background rock was between 8.29 and 89.04%. It contained elements including C, N, O, Na, Al, Si, Cl, Ca, S, K, Ti, Mg and Fe, with a large proportion of C, O, Al, Si and K. The results of SEM showed that these organic matters were attached to surface of minerals. Table 4 and Table 5 only showed samples with C element content greater than 40%.

**Table 4:** EDS results (Element C > 40%)

Source	Elemental quality (%)										EDS results
	C	N	O	Na	Al	Si	Cl	S	K	Ca	
Mozigou, Danba County	40.83	15.4	34.67	2.02	1.00	3.50	1.27	0.23	1.1		Shown in Fig. 8a
Muerzong Township, Maerkang City	44.10		37.63	2.98	1.41	2.73	6.43		3.29	1.44	Shown in Fig. 8b
Longerjia Township, Maerkang City	41.03		42.06	3.19	5.74	5.24	1.96		0.78		Shown in Fig. 8c

**Table 5:** Analysis results of EDS characteristics with organic carbon content greater than 40%

Sample number	C (%)	N (%)	O (%)	Na (%)	Al (%)	Si (%)	Cl (%)	Ca (%)	S (%)	K (%)	Ti (%)	Mg (%)	Fe (%)
DBM-3-018	40.83	15.40	34.67	2.02	1.00	3.50	1.27		0.23	1.10			
DBM-1-002	45.55		40.78	3.11	1.64	3.95	2.77	0.64		1.56			
DBM-1-003	50.02		27.94	2.12		1.37	5.30	0.92		2.46			
DBM-1-006	49.10		32.80			3.49	4.85	4.26		5.50			
MEK-b-1-003	44.10		37.63	2.98	1.41	2.73	6.43	1.44		3.29			
MEK-b-1-004	40.86	9.28	37.92	3.54	0.52	1.62	3.56	0.78		1.92			
MEK-b-3-001	89.04				2.68	2.90						1.56	3.82
LEJ-2-003	41.03		42.06	3.19	5.74	5.24	1.96			0.78			

*DBM* Mozigou, Danba County, *MEK* Muerzong Township, Maerkang City, *LEJ* Muerzong Township, Maerkang City

### Spatial analysis of organic carbon storage in background rocks

According to observation of casting thin section, the reservoir space of rock mainly included intergranular pores, intragranular pores, intercrystalline pores, intracrystalline pores, tectonic fracture, jointed cracks and a small number of dissolved pores. Most of the pores and cracks were semi-filled or completely filled with dark organic matter. SEM and EDS showed that organic matter was not only filled in pores and cracks, but also attached to the mineral surface, indicating that background rocks were rich in organic matter. Porosity test results were present in Table 6.

**Table 6:** Porosity analysis results

No.	Sample ID	Porosity (%)
1.	LEJ-1	2.1
2.	LEJ-2	2.5
3.	GED-1	1.3
4.	GED-2	0.9
5.	DBMZ-1	1.9
6.	DBMZ-2	1.5

*LEJ* Longerjia Township, Maerkang City, *GED* Gaoerda Village, Maerkang City, *DBMZ* Mozigou, Danba County

**Fig. 9:**
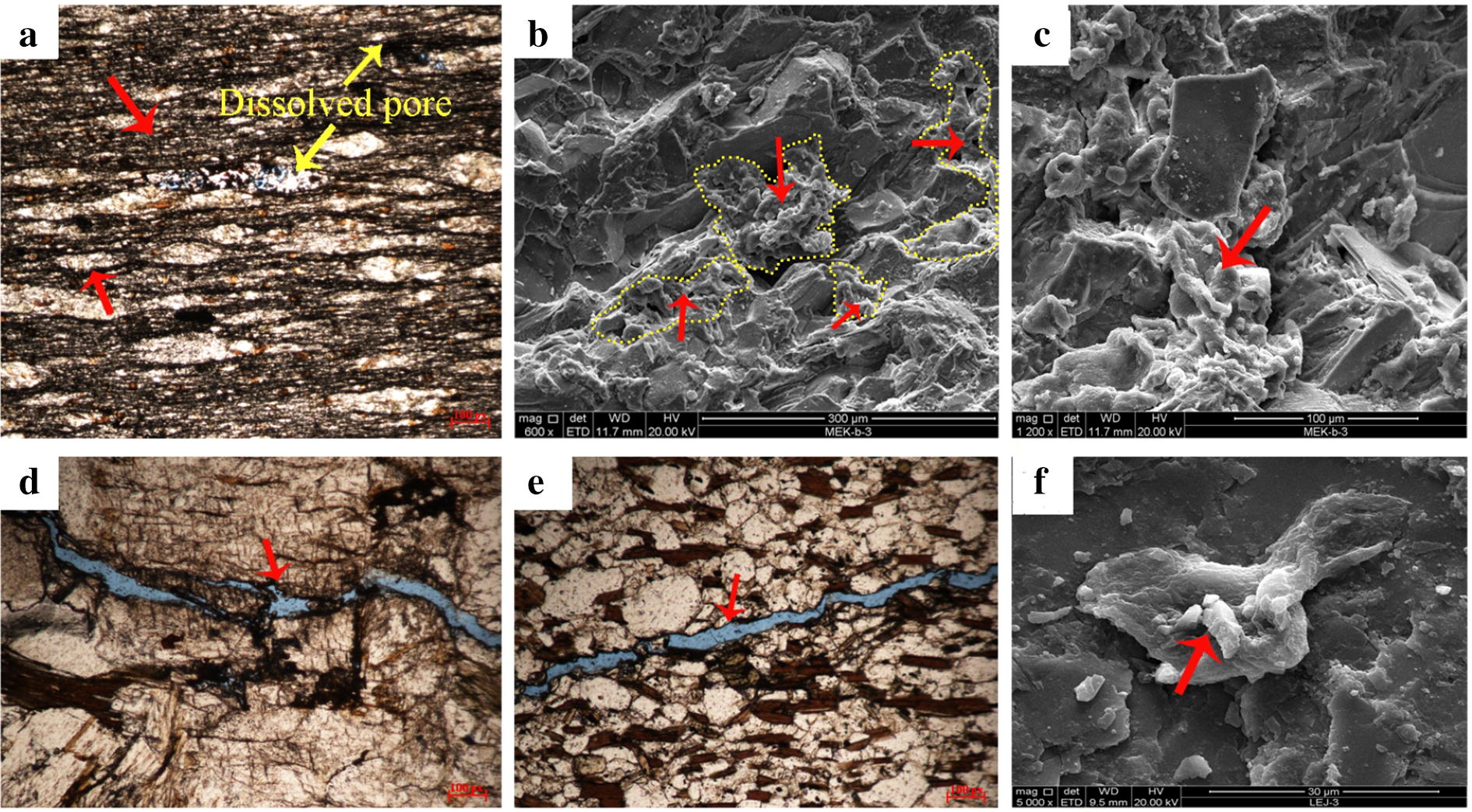
Development of intergranular pores in Maerkang City, Aba Autonomous Prefecture. **a** Longerjia Township, dissolved pore in grains. **b** Baiwan Township, intercrystalline pore and dissolved pore in grains. **c** Baiwan Township, organic matter filling intergranular pore. **d** Muerzong Township, structural fractures and intercrystalline dissolution pore. **e** Baiwan Township, structural fractures and intergranular pores. **f** Baiwan Township, organic matter attached to the mineral surface

**Fig. 10:**
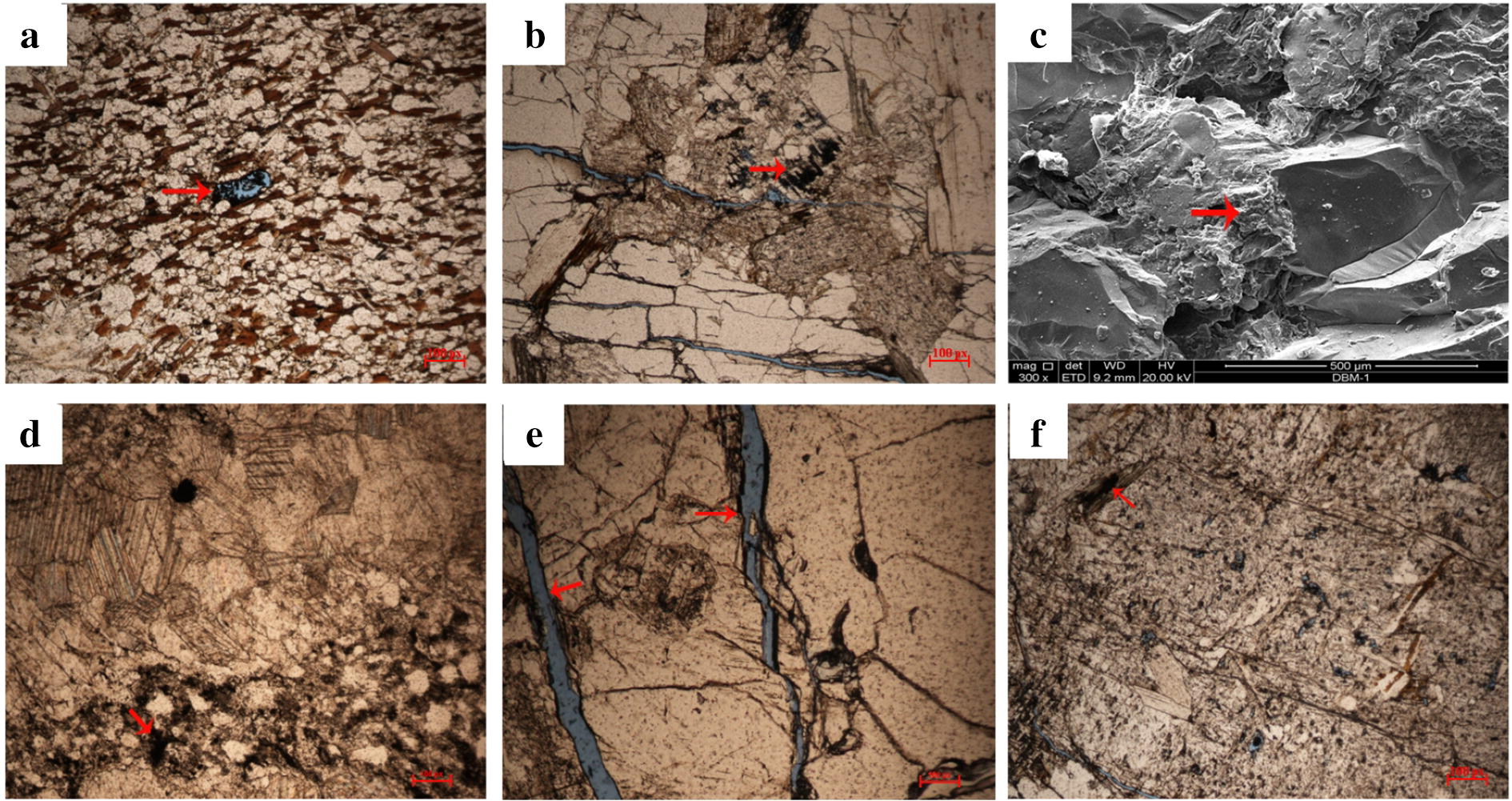
Denudation pores development and cracks, joints. **a** Baiwan Township, Maerkang City, mica denudation. **b** Muerzong Township, Maerkang City, feldspar denudation. **c** Fubian Township, Xiaojin County, denudation. **d** Fubian Township, Xiaojin County, organic matter filling denudateon pore. **e** Muerzong Township, Maerkang City, crack. **f** Mozigou, Danba County, joint development, filled with black organic matters

The intergranular pores and dissolved pores were organic storage pores, both of which were filled with visible organic matter. The cracks and joints were not only reservoir space, but also transport channel for organic matter. This result showed that background rocks of Shilajit were capable to storage and transport organic carbon. The background rock may be the original source of Shilajit exudation, which was storage place of organic matter in Shilajit. As shown in Figs. 9 and 10.

## Discussion

According to the dynamic characteristics of Shilajit exudation area, the paleogeographic environment of sedimentary tectonic structure was Triassic tectonic paleogeographic pattern, which belonged to the residual ocean basin (OB)-spreading ridge (Sr) environment in ocean basin. The sedimentary environment of Triassic was characterized by gradual evolution of shelf slopes and shelf ridges, semi-deep sea slope valleys and slope fans (skirts) in terrigenous clastic shallow sea. At the end of the Triassic, due to collisional orogeny on north side, the ocean basin was closed. The paleogeographic features in the area were transformed into intracontinental environment, which was transformed into Late Triassic foreland basin and developed a thick turbidite system. The sedimentary environment of deposits was alluvial fan-river facies, and the latter was dominated by reticulated rivers. The Yanshanian medium-acid magmatic intrusive activity was strong, and the late Yanshanian intrusive rock was mainly distributed in the stress concentration area or regional fault activity zone [33].

The stratigraphic sequence of Shilajit exudation area was incomplete. While the Triassic strata were mainly distributed in large areas in sedimentary basins, and the Triassic rock combination was dominated by thick semi-deep sea turbidites and contourite (sand slate). In the early and late periods, some distant Yuanbin mudstone, siltstone and sandstone were distributed. After entering the Cenozoic, when marine environment was over, a small fault basin accumulation was formed, representing by river glutenite, siltstone and mudstone combination, and river–lake-phase coal-bearing clastic rock combination.

According to the geological and historical background, combined with the analysis of the research results, the formation mechanism of Shilajit was somewhat complicated. There were several possibilities, which need further confirmation by geochemical research.

- The Shilajit organic matter in the rock formation was evolved from the remains of paleontology. The organic matter was formed in the early Triassic marine layered environment. However, at the end of the Triassic period, the paleogeographic features in the distribution area were transformed from the marine environment to the intracontinental environment, which changed the original environment of high temperature and high pressure, preventing the organic matter from continuing to evolve.
- It may be thermally evolved from mudstones and muddy sandstones adjacent to the mother rock. After the Triassic, the distribution area was mainly the fold uplift period, and the burial heat evolution was basically eliminated, mainly due to the invasion of the granite slurry, resulting in thermal evolution. For example, the shale content of the exudation points of Longerjia Township, Maerkang City was relatively higher. Mainly argillaceous sandstone and sandy mudstone, some organic matter was attached to the mineral surface, which had certain similarities with the oil and gas enrichment on the surface of shale minerals. Therefore, the Shilajit organic matter may be derived from organic rich mudstone.
- It may also be derived from granitic magmatic differentiation. Magmatic activity was closely related to hydrocarbon accumulation and mineralization. The Songpan–Ganzi terrane after the large-scale Indosinian orogeny was affected by the remote effect of the Indian-Asia collision [34, 35]. The Indosinian granitoids (Paleozoic strata and Neoproterozoic crystalline basement) were widely exuded from the Maerkang-Daba sub-terrane (Main exudation area of Shilajit) in the northeast and the Yajiang-Muli sub-terrane in the southwest, these emplacement granitoids were produced by the dome group, which are characterized by zonal distribution in the near north–south direction. Such as the Danba Dome Group and the Muli Dome Group [36]. The main exudation zone of Shilajit had a certain coupling relationship with the spatial distribution of the early Mesozoic granite and the derived pegmatite emplacement, especially in the Maerkang-Daba sub-terrane. The Shilajit exudation points were mostly located in the northeast and southeast of Songpan-Ganzi, where strong magmatic action was observed.

## Conclusion

In this study, it was found that Shilajit mainly distributed on sunny steep slopes and cliffs with a slope of 60° or above at altitude of 2000–4000 m. The control structure surface of the exudation points included rock layer, joint, fracture surface, fault control, and it developed into micro-geomorphology such as concave cavity, wind erosion hole and steep cliff.

The lithology character of the Shilajit exudation area were mainly various metamorphic rocks of sedimentary rocks and a small amount of granite in the Yanshanian period that were rich in organic carbon. Some of rocks developed into intergranular pores, dissolution pores, cracks and joints for storing and transporting organic matter. The organic matter in Shilajit was found to flow out naturally from rocks along pore, structural plane and even accumulate on the surface of rock as a result of storage environment (temperature, stress, structural surface) change caused by rock tectonic action. Further geochemical research is required to confirm the source of organic matter in rocks.

## Data Availability

All data used to support the findings of this study are available from the corresponding author upon request.

## Abbreviations

SEM: Scanning electron microscopy
EDS: Energy dispersive spectrometer
TOC: Total organic carbon
